# Big Data Impacting Dynamic Food Safety Risk Management in the Food Chain

**DOI:** 10.3389/fmicb.2021.668196

**Published:** 2021-05-21

**Authors:** John A. Donaghy, Michelle D. Danyluk, Tom Ross, Bobby Krishna, Jeff Farber

**Affiliations:** ^1^Corporate Operations – Quality Management (Food Safety) Société des Produits Nestlé S.A., Vevey, Switzerland; ^2^IFAS Food Science and Human Nutrition, University of Florida, Gainesville, FL, United States; ^3^Centre for Food Safety and Innovation, University of Tasmania, Hobart, TSA, Australia; ^4^Department of Food Safety, Dubai Municipality, Dubai, United Arab Emirates; ^5^Department of Food Science, University of Guelph, Guelph, ON, Canada

**Keywords:** data, food, safety, risk, management

## Abstract

Foodborne pathogens are a major contributor to foodborne illness worldwide. The adaptation of a more quantitative risk-based approach, with metrics such as Food safety Objectives (FSO) and Performance Objectives (PO) necessitates quantitative inputs from all stages of the food value chain. The potential exists for utilization of big data, generated through digital transformational technologies, as inputs to a dynamic risk management concept for food safety microbiology. The industrial revolution in Internet of Things (IoT) will leverage data inputs from precision agriculture, connected factories/logistics, precision healthcare, and precision food safety, to improve the dynamism of microbial risk management. Furthermore, interconnectivity of public health databases, social media, and e-commerce tools as well as technologies such as blockchain will enhance traceability for retrospective and real-time management of foodborne cases. Despite the enormous potential of data volume and velocity, some challenges remain, including data ownership, interoperability, and accessibility. This paper gives insight to the prospective use of big data for dynamic risk management from a microbiological safety perspective in the context of the International Commission on Microbiological Specifications for Foods (ICMSF) conceptual equation, and describes examples of how a dynamic risk management system (DRMS) could be used in real-time to identify hazards and control Shiga toxin-producing *Escherichia coli* risks related to leafy greens.

## Introduction

An estimated 600 million people fall ill through the consumption of contaminated food and 420,000 die every year, resulting in the loss of 33 million Disability-Adjusted Life Years (World Health Organization, 2015). These estimates can be used to direct food safety policy and risk management options (Scallan et al., 2011).

The need for a risk-based approach for production of safe food is underpinned by the adoption of HACCP with the necessary prerequisite programs. In turn, risk management metrics, with tools and concepts such as Food Safety Objectives (FSO) and Performance Objectives (PO), proposed by the International Commission on Microbiological Specifications for Foods (ICMSF) and adopted by the Codex Alimentarius Commission (International Commission on Microbiological Specifications for Foods, 2010; Codex Alimentarius Commission, 2016) represent a more quantitative risk management approach that can be used by intergovernmental agencies, national governments, and food businesses. Food safety related data, acquired throughout the food chain is required for real-time food safety decision-making by all stakeholders, including risk assessors, managers, and communicators.

Global megatrends, described as transformative global forces will pose significant challenges to future global food safety, food security, and nutrition (High Level Panel of Experts, 2017; King et al., 2017). Key drivers for change include: Global economic growth/investment/trade pricing, innovation in food production and productivity, structural and socio-economic impacts on food supply chains and changing food safety and quality management systems.

The rapid expansion of the Internet of Things (IoT) and digital transformation (Wellness Recovery Action Plan, 2015; Food and Agriculture Organization and World Health Organization, 2018) has enabled the collection and transfer of “big data,” in real-time. This can impact most parts of the food chain. Key characteristics of big data are often referred to as: volume of data produced; velocity – speed of data streaming; veracity – uncertainty of data; variety – structured and unstructured data; and ultimately value – the worth of data for actionable insights and information for food safety, public health, and trade (Marvin et al., 2017). The ability to collect, analyze, and convey digital data at all stages of the food value chain has seen an exponential increase in the volume, velocity, variety, and veracity of data available. Data sources impacting food safety include food production, food consumption, public health, agriculture, environmental conditions logistics, social media, etc., containing structured and unstructured formats. The ability to extract value from these data, while ensuring the interoperability of different sources to assure food safety and quality, is the future challenge. While data scientists can easily build tools and dashboards to visualize data, often the outcomes have highlighted the need to have “clean” and reliable (meta)data, in formats that can be accurately interpreted by subject matter experts (Ridzuan and Zainm, 2019).

The emergence of precision agriculture, often referred to “smart farming,” using digitalization in farming practices (Bhakta et al., 2019; Shafi et al., 2019) and “omics-based” precision food safety (Kovac et al., 2017; Kovac, 2019) leverages linked data for foodborne outbreak traceability, predictive analytics, artificial intelligence (AI) and machine-learning in the realm of food safety management. Digital transformation in food manufacturing and supply chain enables better utilization of data for more dynamic risk management, automated adjustment for deviations, real-time transparency of food safety and quality control parameters, product release, defect rate reduction, and trend analysis.

This paper examines the increasing opportunities to collect, integrate, analysis, and interpret data throughout the food value chain, to predict, assess, and manage microbial food safety risks, in the context of a dynamic risk management system (DRMS). Simulation scenarios are described whereby Shiga toxin-producing *Escherichia coli* (STEC) contamination of leafy greens occurs during production and processing, but it can be controlled in real-time by using big data to investigate and manage the risk. The challenges of valuable data availability, accessibility, ownership, and interoperability will also be discussed.

## Precision Agriculture and Digital Food Supply Chain: Inputs for Food Safety Management

Food production, and concomitantly the safety and quality of food, originates on farms. Increased data availability *via* the internet now allows for devices on farms to connect to IoT networks to collect and interpret data from the beginning of the supply chain through to retail/consumers. Precision agriculture enables agricultural production systems to deploy robotics, sensors, global navigation satellite systems (GPS), and big data analytics to gather unique data on a more precise, spatial, and temporal scale (Aqeel-Ur-Rehman et al., 2014; Wolfert et al., 2017; Weersink et al., 2018; Astilla et al., 2019). Consequently, the information gathered can be used for the application of more prescriptive inputs to support production crop/livestock yields, environmental impacts, economic returns, and food security/safety to improve food safety management decisions. Kamilaris et al. (2017) provide an example of data that can be garnered from precision agriculture to help guide food safety, albeit not specifically microbiological safety.

Using the tools of smart farming and data analytic platforms, the needs of individual livestock or crop areas can be targeted and customized with inputs such as rotation feeding, pesticide application/withdrawal or irrigation (Ramundo et al., 2016; Grieve et al., 2019). Furthermore, these technologies can provide an opportunity for farmers to increase food security with decreased environmental impact (Garnett et al., 2013).

A major benefit of the emerging agricultural precision technologies is the opportunity to ensure greater transparency, traceability, provenance as well as food safety and quality attributes (Danezis et al., 2016). Investigations of foodborne outbreaks linked to plant or animal-based products (Currie et al., 2019; Self et al., 2019), food fraud (Van Rijswijk and Frewer, 2012), and consumer demand for food provenance (Badia-Melis et al., 2015; Danezis et al., 2016), confirm the need for assimilation and integration of “agricultural” data.

The future prospect is that big data can be used to predict the presence of pathogens or contaminants, by linking environmental information with pathogen growth and/or hazard occurrence. For example, by monitoring the conditions of crops in the field including weather data, the areas with an increased potential of aflatoxins can be identified before the crop enters the food chain (Armbruster and MacDonell, 2014). Environmental informatics can help identify high-risk periods, which may trigger effective control for the downstream food supply chain. Data gathered from digital information systems on farm, including field scanning drones, can precisely identify areas within a field, which are subject to aflatoxin contamination because of particular crop conditions. In turn, this real-time data, facilitated by smart technologies including AI, directs mitigation measures, in the form of dynamic harvesting, to prevent food quality issues downstream (Grieve et al., 2019). Drones, which are now regularly used in large-scale agriculture, will be increasingly used to harness data related to food safety and quality, e.g., animal intrusion in crop fields or localized field flooding conditions (Cancela et al., 2019).

Potential also exists for large farm owners to use wireless IoT applications to collect data regarding the location, well-being, and health of their cattle (Busse et al., 2015; Köksal and Tekinerdogan, 2019). Biosensors and wearable technologies may be used to identify unhealthy animals (Neethirajan, 2017; Vidic et al., 2017). Availability of such real-time data enables livestock managers to separate unhealthy animals from the herd. In some circumstances, such herd/flock management could mitigate potential food safety issues in the human population e.g., if the carrier/shedder status of both healthy and unhealthy animals, was available for foodborne pathogens. Strawn et al. (2013) have described the predictive potential of combining environmental and meteorological data for the presence of *Listeria monocytogenes*.

It is foreseeable that further data collecting devices and databases will be interconnected to provide voluminous structured and unstructured data at the farm level. This will be used to enhance source attribution analysis in foodborne outbreak scenarios and enable more precise farming for food safety and quality attributes. The data sources that may be used include: crop worker health status, water quality and usage, meteorological information, real-time livestock health status, animal health records, veterinary medicine prescription records, animal movement records, animal feed quality and usage, and farm audit certification records. However, it must be acknowledged that some data, e.g., related to personnel (health/medical status) will be inaccessible and therefore data indicative of GAP/hygienic practices may be more readily available.

There are still many processes that are done manually in the food industry, but this can change with the increasing use of IoT driven food processing equipment (Bayano-Tejero et al., 2019), and the use of IoT in water dispensing, cleaning and disinfectant dosing, pest monitoring, etc. Automation can reduce repetitive processes, making them more efficient and the digital contents and data generated, can provide better insights about the day-to-day operations (Hasnan and Yusoff, 2018).

Blockchain technology (BCT) is a distributed, decentralized, public ledger (or database) for permanent and verifiable record-keeping that can transform food systems by increasing efficiency, transparency, and collaboration throughout the chain (Antonucci et al., 2019). BCT provides the opportunity to access and use data, shared by connected partners (public and private) in the food supply chain (Sander et al., 2018; Tripoli and Schmidhuber, 2018). It is foreseeable that upstream food safety parameters, collated and logged in BC ledgers could be analyzed and used as part of a risk-based inspection system downstream in the supply chain.

## Precision Public Health/Consumer Feedback: Inputs for Food Safety Management

Precision public health uses surveillance data and sophisticated analytics to accurately measure the global prevalence and consequences of foodborne diseases (Dowell et al., 2016), pathogens in food, and environmental exposure, as well as consumer behavior, in a way that allows better assessment of population health risk factors, and the development of policies and targeted programs (Khoury et al., 2016) to prevent foodborne diseases. Data gathering has helped in public health interventions including use of:

- Publically available data sets and data obtained from social media to predict the occurrence of critical food safety violations in food businesses (Goldsmith, 2015).
- Mobile Call Detail Records (CDR) to track human population movement, while managing outbreaks (Wesolowski et al., 2014; Jones et al., 2018).
- Use of supermarket courtesy cards and e-commerce sales records in outbreak investigations and increasing the effectiveness of product recalls (Møller et al., 2018).

A wide range of structured datasets are currently available or could be accessed for developing improved public health interventions. These include:

- The WHO food safety platform FOSCOLLAB and other National/Regional food safety databases such as TESSY, RASSF (Marvin et al., 2017).
- Electronic healthcare and medical records (Goldstein et al., 2016), medical claims data and OECD pharmaceutical demand data, where accessible without breaches of personnel privacy.
- Certification data (product, management system, personal certification, etc.) available with third-party certification bodies

There are also large sets of high volume, unstructured data that could be used to trace food incidents or exposure to a particular food by analyzing:

- Food consumption patterns and trends that could be obtained from digital food related platforms used for food ordering or diet management
- Social media interactions (e.g., increased requests for information on how to treat diarrhea) that could indicate the prevalence or spread of an illness (Broniatowski et al., 2014).

## Precision Food Safety: Inputs for Food Safety Management

The rapid growth of “big data” in the food industry has revolutionized the field of microbial food safety and has led to the term, “precision food safety,” to describe all the various new data sources that can be used to improve food safety risk management (Kovac et al., 2017; Kovac, 2019). For example, genomics-based approaches based on whole genome sequencing (WGS) are greatly advancing our ability to retrospectively detect food safety outbreaks, and WGS is now being routinely used to characterize human, food, and environmental isolates of all key foodborne pathogens, bringing a high level of precision to interpretation of foodborne disease outbreaks (Ronholm et al., 2016; Brown et al., 2019).

An example of this accumulation of big data is the creation of the United States FDA’s GenomeTrakr WGS Network, which is the first integrated network of state and federal labs to use WGS to track foodborne pathogens to improve outbreak response activities (Allard et al., 2016). The network consists of a publicly available global database containing the genetic makeup of thousands of foodborne disease-causing bacteria from food and environmental sources housed at the National Center for Biotechnology Information (NCBI) at the National Institutes of Health (NIH; Tolar et al., 2019). Participants in GenomeTrakr include not only United States federal and state laboratories, but also international and reference laboratories.

A big advantage of using WGS in an integrated manner is that outbreaks can be identified at an earlier stage and therefore resolved faster (Besser et al., 2019). In fact, WGS can provide information beyond the identity and relationship of strains and can help to improve the safety, quality, and shelf life of foods. This can be extremely valuable for regulators and the food industry to help design, prioritize, and implement effective risk management interventions (Food and Agriculture Organization, 2016). For food manufacturers, an advantage of using WGS would be differentiation of transient and resident pathogens, as part of a food company’s environmental monitoring program. Furthermore, WGS can be used for source attribution related to country of origin (Franz et al., 2016; Koutsoumanis et al., 2019).

In addition to source tracking/attribution, WGS can also (i) enable an assessment of the AMR status of strains; (ii) provide insights into bacterial adaptation and survival; and (iii) as well as provide information on pathogenicity. The latter information can be used, for example, to better inform risk assessments, e.g., assessment of the presence of less pathogenic *L. monocytogenes* strains containing a truncated internalin A protein (Chen et al., 2011; Nielsen et al., 2017).

Omic approaches such as metagenomics are also very valuable to help us to understand the microbial communities (i.e., the microbiomes) and the genes of interest directly from a food sample, without the need for isolation of the specific bacteria that provide those genes (Doyle et al., 2017; Forbes et al., 2017). An ideal approach would be to develop metagenomics methods that are independent of culture and combine both the detection and subtyping of the infecting organisms (including the “natural” microbiome) directly from a food or clinical specimen (Ottesen et al., 2020). In the area of food spoilage, one can use omics technology to develop microbiome-based pathogen control strategies that can be implemented in the food industry. One example is the development of advanced sanitation approaches based on an understanding of the different microbial ecologies found in individual food processing environments (Gu et al., 2019).

The next step toward advancing food safety risk management is using a systems-based and machine learning approach to omics that would integrate all of the genomic, metagenomic, phenotypic, and epidemiological data, with risk assessments informed by these data (Deneke et al., 2017).

## Outbreaks: Potential Uses of Big Data for Dynamic Risk Management

In 2018, two outbreaks of *E. coli* O157:H7 were linked to the consumption of romaine lettuce in Canada and the United States (Centers for Disease Control, 2018, 2019; Public Health Agency of Canada, 2018, 2019). In the first outbreak, clusters of *E. coli* O157:H7 were linked by WGS sequences, and leafy greens from Yuma growing region identified as the likely source (Food and Drug Administration, 2018a). The traceback investigations (Gerrity, 2018) identified a total of 36 growing fields on 23 farms in Arizona and California as potential sources of contaminated lettuce, feeding into seven intermediate shippers. Only one of these shippers did not comingle romaine lettuce from multiple farms and shipped the romaine as whole-head product. Follow-up environmental assessment weeks after the outbreak identified three water samples from an irrigation canal that delivered water to farms in the local area, including several identified, containing *E. coli* O157:H7 with the same WGS genetic fingerprint as the outbreak cases (Food and Drug Administration, 2018b). A concentrated animal feeding operation (CAFO) was located adjacent to the irrigation canal, but no obvious route for *E. coli* O157:H7 contamination was determined, and none of the samples collected contained the outbreak strain. Growers suggested that leaf freeze damage and dew on romaine leaves created conditions favorable for windborne contamination, with dust carrying the outbreak strain. Dust has previously been identified as a potential route for STEC contamination (Berry et al., 2015). The commingling of romaine lettuce from various farm growing fields at fresh-cut produce manufacturing/processing facilities complicated FDA traceback efforts. In the following, 2018–2019 season, FDA sampled 118 romaine lettuces for *E. coli* O157:H7 and *Salmonella* at 26 commercial coolers and cold storage facilities (Food and Drug Administration, 2019a). *Salmonella* was not found, and one sample was positive for a non-pathogenic *E. coli* O157:H7.

In the second outbreak (Centers for Disease Control, 2019; Public Health Agency of Canada, 2019), clusters of *E. coli* O157:H7 were first linked by WGS (Food and Drug Administration, 2019b). WGS also revealed that the *E. coli* O157:H7 strain was closely related to an *E. coli* strain from ill people in a 2017 outbreak linked to leafy greens, but not related to the first, 2018 outbreak discussed above. Traceback investigations initiated from six points of service identified 14 distributors, 17 farms, and 15 specific fields in multiple California counties. This was eventually narrowed to one farm, which was also identified as one of the potential suppliers of leafy greens or romaine lettuce in the 2017 outbreak investigations. A sediment sample from an on-farm water reservoir tested positive for the outbreak strain. While the precise route of contamination is uncertain, plausible ways in which this water may have contaminated the romaine lettuce included direct harvest/postharvest application to the crop and/or to harvest equipment food contact surfaces. While the farm did have a procedure in place to collect and test the reservoir water for generic *E. coli* and treat the water tank with a sanitizer before use, verification procedure records did not document that the water sanitizer was present at levels that would assure that the water was not contaminated with pathogenic bacteria. Inspection of water tank sanitizer treatment systems used revealed that some units had undissolved sanitizer cakes and that some tank systems were constructed in a manner that likely did not allow for optimal sanitizer treatment of the agricultural water before use.

**Figure 1:**
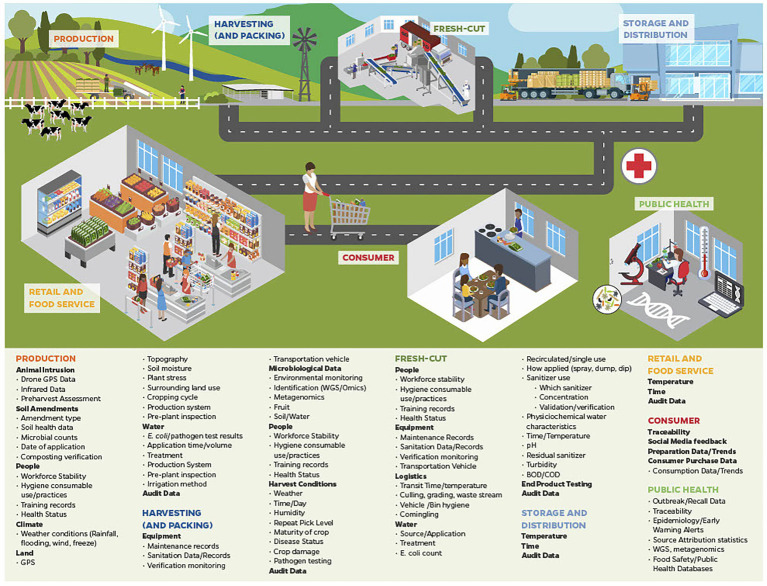
A number of examples of the types of big data currently or potentially available across the leafy green food chain that can be integrated for use in traceback to determine implicated product source during outbreak situations, and can be used to inform dynamic risk management systems (DRMS).

Potential sources of big data that can be generated and collected during leafy green production, such as harvesting, cutting, packing, distribution, retail sale and from consumers are identified in Figure 1. Many of these sources and types of data have been discussed above. Data generated through WGS was able to identify both outbreaks detailed above, and even linked the second outbreak to an outbreak from 2017. It is easy to see how traceability technology (e.g., BCT) information can be linked between production fields, farms, harvesters, distributers, and points of services, and that lengthy and complicated traceback efforts could have been greatly accelerated.

## Microbiological Quantitative Risk Management in a Digital/Big Data Era

The conceptual ICMSF equation proposes that the change in food safety (microbiological) risk from a food can be understood in terms of the potential for growth and/or recontamination (“ΣI”), or inactivation (“ΣR”) of relevant pathogens in the food as they move through the supply chain and final processing of the food prior to serving and consumption. It is usually expressed as:

$$H_{o}-\Sigma R+\Sigma I\leq \mathrm{FSO}$$

and is based on the final level of contamination at the point of consumption.

The equation proposes that the sum of the processes during the farm-to-fork continuum that allow pathogen growth, minus those processes in the same pathway that involve pathogen inactivation, determine the risk of illness to consumers, because they estimate the average levels and prevalence of pathogens in the product at the time of consumption.

**Figure 2:**
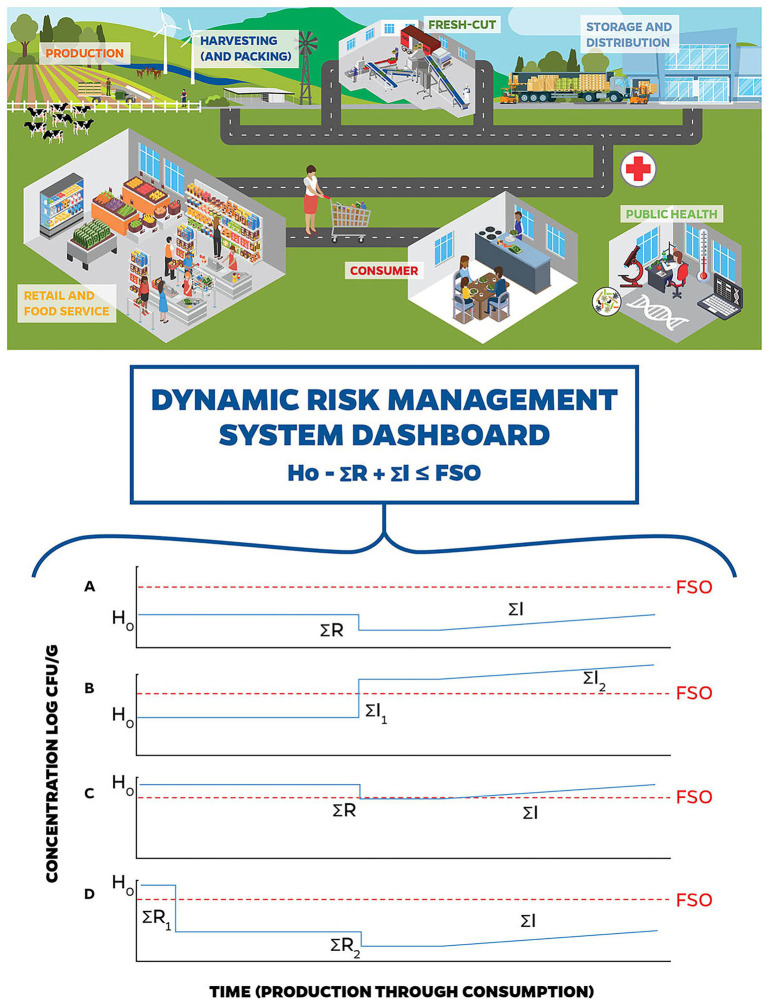
Stylized DRMS. Dynamic risk management involves the use of big data for quick identification of changing conditions resulting in potential changes to the hazard analysis and facilitates decision-making to manage those risks in real-time. **(A)** through **(D)** represent examples of how a DRMS could have been used to identify hazards and control Shiga toxin-producing *Escherichia coli* (STEC) risks related to leafy greens. **(A)** represents the baseline conceptual risk management in a fresh-cut leafy greens operation, where the initial hazard level, controlled by preventive measures during production is below the Food Safety Objectives (FSO), and (i) no introduction or cross-contamination occurs during harvest; (ii) there is a small reduction during washing, and (iii) potential increases in STEC populations are controlled by the cold chain. **(B)** represents STEC contamination of leafy greens during processing. The initial hazard level is controlled by preventive measures during production, and (i) no introduction or cross-contamination occurs during harvest, (ii) untreated water used during the wash step introduces a high level of STEC, and (iii) potential increases in STEC populations are controlled by the cold chain. In this case, DRMS would have had the real-time data surrounding the wash water (see Figure 1), flagged a concern, and allowed for risk management decision-making that could have prevented entry of this product into the supply chain. **(C)** represents STEC contamination of leafy greens during production. Due to a breakdown in pre-harvest preventive measures, the initial hazard level is higher than the baseline, which may be due to a combination of factors including (i) proximity of concentrated animal feeding operations (CAFOs), (ii) unusual weather patterns (i.e., winds and freezing), and/or (iii) contaminated water sources used in applications that contacted harvested product. For this scenario, (i) no introduction or cross-contamination occurs during harvest; (ii) there is a small reduction during washing, and (iii) potential increases in STEC populations are controlled by the cold chain. In this example, DRMS would have the real-time data around production practices (see Figure 1), flagged a concern, and allowed for risk management decision-making to prevent entry of this product into the supply chain. **(D)** represents a potential response to **(C)** using DRMS. When DRMS flagged a concern with production water use (see Figure 1), the risk management decision is made to treat the water (e.g., chemical treatment, UV light) that contacts the harvestable portion of the leafy greens (i.e., overhead irrigation, foliar sprays, and aerial sprays) prior to application to reduce the introduction of STEC from this source. The risk management decision to treat the irrigation water decreases STEC levels to below the FSO.

Accordingly, if we can estimate the overall potential for pathogen growth, compared to pathogen inactivation in a food/food process, we can begin to estimate the risk of illness to a consumer. The ICMSF equation encapsulates these considerations by evaluating the initial level of contamination («H_o_»), the potential for increase (growth and recontamination) or cross contamination («ΣI»), and the potential for inactivation («ΣR»), all measured in log CFU. The potential for dynamic risk management to identify hazards and control STEC risks related to leafy greens using big data inputs to elements of the ICMSF conceptual equation is represented in Figure 2.

The potential for sensors and communications technologies, integrated with predictive microbiology models to assess the microbiological quality and safety of foods in domestic and international supply chains has already been demonstrated (Goldstein et al., 2016; Vidic et al., 2017). This is an example of big data being used to improve the microbiological quality and safety of foods.

## Challenges Beyond Opportunities

An integrated system incorporating digitally-derived “big data” as inputs for a dynamic food safety risk management, remains a distinct possibility. The opportunity to use unused data sources, beyond classical food safety management data (e.g., critical limits in HACCP, process data, chill chain parameters, etc.) can impact not only transparency and traceability, but also can be used to predict and anticipate higher risk scenarios as well as plan and implement mitigation measures for microbiological food safety issues.

However, the digitally disruptive era is not without its challenges in the food safety realm. Foremost is the availability and accessibility to internet connectivity across increasingly global food value chains. Furthermore, there is the technical infrastructure and perceived benefit discrepancy between developing and developed countries, large food manufacturers and Small Medium Enterprises (SME) to consider.

While the volume and velocity of data from precision agriculture, precision public health, and precision food safety increases exponentially, the variety and veracity, *viz a viz*, mismatch between provider and end user, increasing extent of unstructured, unverified and biased user population data sources e.g., social media, blogging, will require a form of validation, cleansing or normalization of the data to avoid distortion or misrepresentation. Furthermore, transparency, confidentiality, accuracy, and fairness are important aspects of responsible data science (Responsible Data Science, 2016).

Data scientists will play an increasingly important role in collation, combination and visualization of different data sources, while subject matter experts such as microbiologists, will still have a vital role to assess, challenge, and interpret this collated data for proper risk management and communication.

While technical issues remain on the interoperability and interconnectivity of the IT sources (Manyika et al., 2015), greater hurdles exist around the ownership, governance, sharing, accessibility, and security of the derived data. For example, concern has been raised on the contractual issues relating to precision agriculture, where larger companies invest in digital collection systems, which may exclude many stakeholders with valuable input to food safety management (Carbonell, 2016).

A key tool in precision food safety is WGS, however, inaccessibility to private and some public sequenced pathogen databases, while understandable from some stakeholder perspectives (Jagadeesan et al., 2019), may limit dynamic risk management assessment/options. Similarly, uptake of BCT requires minimum data standards with practical and user-friendly interphases between all parties, if it is to be widely adopted in both the private and public sector. The perceived bureaucracy and value to smaller stakeholders in the foodchain are deterrants to contribute to BCT and therefore garbage in garbage out (GIGO) can be the consequence.

Near real-time interventions, for the management of food safety risks, based on the diversity of data throughout the value chain, will require validation using numerous iterations of relevant data, related to case studies for foodborne outbreak/cases. Validation is particularly important, as these types of data are increasingly being used as the basis for regulatory actions, civil suits and even criminal cases. A court challenge that indicates that the science could not be totally relied upon would set a legal precedent that greatly reduces its potential role as a driver.

An avalanche of data – so-called infodemic, as illustrated by the Covid 19 pandemic, from verified scientific sources and unverified data, can disrupt the role of the risk manager. Therefore, risk assessors, dealing with such data, must use it cautiously to inform risk assessment, while ultimately, the decision making regarding food safety, rests with risk managers.

Furthermore, the cost and technical expertise needed to achieve precision food safety systems is likely to increase. It is unlikely that developing countries are going to be able to achieve the degree of infrastructure needed, at least in the short term. This will further aggravate the inability of developing countries to gain entry to the markets of developed countries, an issue already of concern internationally. This is particularly important when one considers that export of agricultural commodities and specialty crops is one of the primary ways that developing countries gain access to “hard currencies,” which is critical to developing country economic health.

## Data Availability Statement

The original contributions presented in the study are included in the article/supplementary material, further inquiries can be directed to the corresponding author.

## Author Contributions

All authors as members of the International Commission on Microbiological Specifications for Foods (ICMSF) contributed equally to the ideation, scoping, and writing of the paper. The paper contents were agreed upon and approved by all members of the ICMSF. All authors contributed to the article and approved the submitted version.

### Conflict of Interest

The authors declare that the research was conducted in the absence of any commercial or financial relationships that could be construed as a potential conflict of interest.
